# Supplementary material for: Tim-3 Coordinates Macrophage-Trophoblast Crosstalk via Angiogenic Growth Factors to Promote Pregnancy Maintenance
Source: Int J Mol Sci. 2023 Jan 12;24(2):1538. doi: 10.3390/ijms24021538 (PMC9867110; doi:10.3390/ijms24021538)
Supplement: Supplementary file 1 [file ijms-24-01538-s001.zip › ijms-2056464-supplementary.pdf]

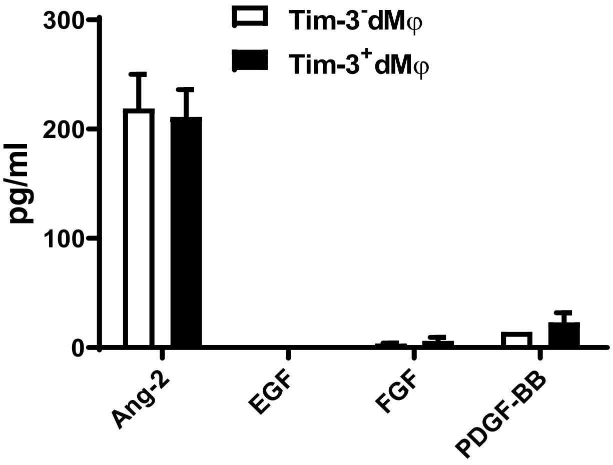

Figure S1. The levels of angiogenic growth factors in supernatants of Tim-3^{-}dMφs and Tim-3^{+}dMφs. The levels of Angiopoietin-2 (Ang-2), EGF, FGF, PDGF-BB in supernatants of Tim-3^{-}dMφs and Tim-3^{+}dMφs was evaluated using Multi- Analyte Flow Assay Kit. Data represent mean ± SEM.
